# Delta9-tetrahydrocannabinol: A blunt weapon or a double-edged sword for virus-induced neuroinflammation

**DOI:** 10.4103/NRR.NRR-D-25-01394

**Published:** 2026-01-02

**Authors:** Alison R. Van Zandt, Miranda D. Horn, Andrew G. MacLean

**Affiliations:** Tulane National Biomedical Research Center, Tulane University, Covington, LA, USA; Biomedical Sciences Training Program, Tulane University School of Medicine, New Orleans, LA, USA; Department of Biological Sciences, Neuroscience Program, Ohio Wesleyan University, Delaware, OH, USA; Department of Microbiology and Immunology, Tulane University School of Medicine, New Orleans, LA, USA; Tulane Brain Institute, New Orleans, LA, USA; Louisiana Cancer Research Center, New Orleans, LA, USA; Tulane Center for Aging, New Orleans, LA, USA

Delta9-Tetrahydrocannabinol (Δ^9^-THC), the primary psychoactive component of cannabis, has demonstrated both neuroprotective and anti-inflammatory properties, while also having the potential to impact the blood–brain barrier and cognitive function with chronic use. A previous study hosted at Tulane National Biomedical Research Center showed that chronic Δ^9^-THC administration prior to simian immunodeficiency virus (SIV) infection reduced viral load and generalized inflammation, including in the cerebellum (Molina et al., 2011). Although the effect of Δ^9^-THC on reducing microglial activation has been explored in the context of chronic human immunodeficiency virus (HIV) infection, the influence of Δ^9^-THC on the initial seeding of the central nervous system (CNS) reservoir, reservoir persistence, and downstream neurodegeneration remains largely unknown. This perspective explores the impact of cannabinoids on HIV neuropathology with a focus on regenerative potential.

**Figure 1:**
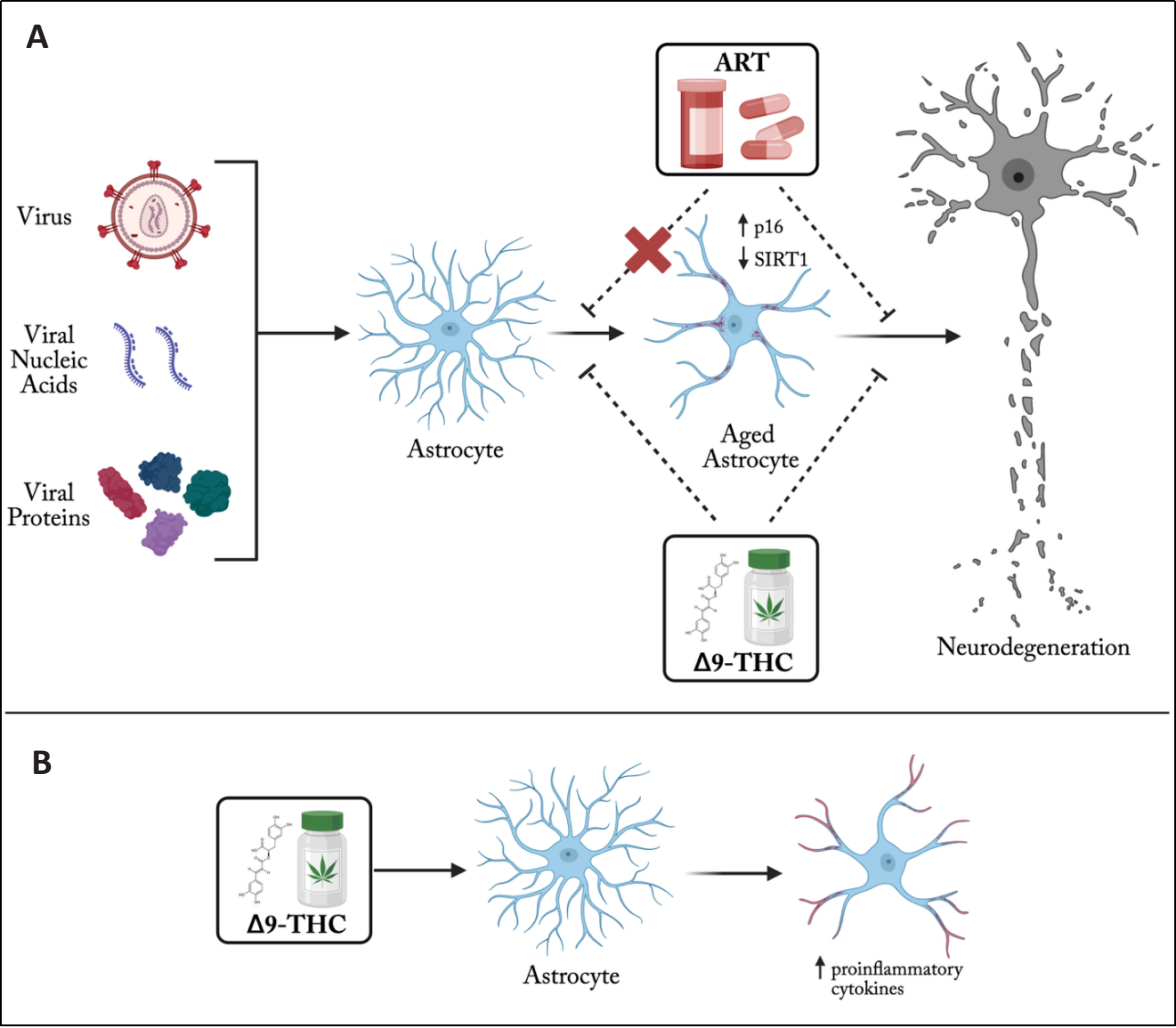
Schematic for the overall hypothesis. (A) Viral proteins and nucleic acids induce chronic inflammation and an aged phenotype in astrocytes (increased p16, decreased sirtuin 1 (SIRT1) function, and increased glial fibrillary acidic protein combined with downstream neurodegeneration. Combination antiretroviral therapy (ART) partially prevents this process. Our hypothesis is that delta9-tetrahydrocannabinol (Δ^9^-THC), as a synergistic adjunct to combination ART, would prevent the altered astrocyte phenotype, the neurodegeneration, and cognitive decline. (B) In a healthy environment, Δ^9^-THC alone can activate astrocytes toward a proinflammatory phenotype characterized by increased cytokine secretion. Our hypothesis is that Δ^9^-THC functions as a bidirectional modulator, protective in the presence of ART during HIV infection yet capable of promoting glial activation under non-infectious conditions. Created in BioRender. Van Zandt, A. (2025) https://BioRender.com/di031sd.

**Virus-induced inflammation leads to senescence and neurodegeneration:** Infiltrated inflammatory leukocytes in key brain regions are central to HIV-associated neuroinflammation, making glia a primary target for modulating downstream neurodegeneration and associated cognitive decline. Activated microglia and astrocytes are associated with increased leukocyte transmigration, increased HIV infection of the brain, and the establishment of a highly inflammatory environment. Together, these features further aggravate HIV-associated disease mechanisms, including neurodegeneration (**Figure 1**). Δ^9^-THC has known anti-inflammatory properties, which could inhibit the inflammation observed. Cannabinoids could reprogram leukocytes and glial cells to less inflammatory phenotypes that would reduce initial seeding of the CNS and later persistence of the reservoir. Indeed, it is possible that Δ^9^-THC could even prevent the cycle of neurodegeneration and cognitive decline from starting in the first place.

Chemotaxis of monocytes and T cells increases during acute S/HIV infection and, following the initial neuroinvasion by these leukocytes, there is activation of glia and generalized neuroinflammation. Multiple rounds of CNS seeding are highly probable, potentially driven by viral infection and associated inflammation within the CNS. Our recent study shows an upregulation of inflammatory and senescent makers in several brain regions in SIV-infected rhesus macaques, which is associated with neurodegeneration and only partially diminished by antiretroviral therapy (ART) in specific brain regions, including the hippocampus (Horn et al., 2025). This suggests there is ongoing glial dysfunction driving the neurodegenerative process. In turn, this neurodegeneration is likely to have region-specific effects on cognition and behavior as seen in HIV-associated neurocognitive disorders. The mechanisms by which neurodegeneration, cognitive decline, and blood–brain barrier dysfunction persist despite ART-suppression – and whether these effects could be reversed by Δ^9^-THC treatment – remain unknown, representing a critical gap in our knowledge.

**Reversal of virus-induced senescence with delta9-tetrahydrocannabinol:** The role of Δ^9^-THC in preventing the dysregulation of leukocyte and glial function and associated neuroinflammation, especially in the context of successful ART suppression of virus, represents an active field for investigation. There is a critical need to understand the mechanisms whereby Δ^9^-THC prevents or even reverses glial activation, neuroinflammation, and downstream neurodegeneration, which is what our recent study attempted to answer (Van Zandt et al., 2025). While some aspects of virus-induced senescence were inhibited by chronic Δ^9^-THC administration *in vivo*, Δ^9^-THC could not reverse senescence when added to cultured cells from SIV-infected macaques *ex vivo* (Van Zandt et al., 2025).

While the complex interplay of immune cells contributing to CNS inflammation in HIV is recognized – involving infiltrated leukocytes, resident microglia, and astrocytes (Horn et al., 2025) – the pivotal role of drugs of abuse as key drivers remains largely unexplored. Specifically, the mechanisms by which Δ^9^-THC influences: (1) the initial seeding of viral reservoirs within the CNS prior to ART initiation, and (2) the orchestration of chronic neuroinflammation and neurodegeneration through intercellular communication, are critical unknowns. Understanding the role of Δ^9^-THC on these processes is crucial for advancing our understanding of the cellular mechanisms underlying HIV-associated neurocognitive disorders and potential therapeutic targets.

Prior analyses have noted chronic activation in the CNS of people living with HIV (PWH), SIV-infected rhesus macaques, humanized rodents, and cells in culture exposed to virus and viral proteins. Notably, while a prior study demonstrated increased cognitive decline in chronic SIV infection with morphine (Marcario et al., 2016), they were not specifically designed to determine if changes in cognition correlated directly to inflammation, nor did they assess the effects of ART or Δ^9^-THC. However, dissecting out the effects of peripheral viral infection with how infection of the CNS is initiated is limited, especially in the context of concurrent oral Δ^9^-THC, and only Δ^9^-THC, use.

Cannabinoids also alter the dynamics of HIV infection. Positron emission tomography imaging in virally-suppressed PWH demonstrates that the number of activated glia in the CNS of PWH is inversely correlated with cognitive performance (Rubin et al., 2018). Cannabis-using PWH display equal or lower viral load and circulating HIV nucleic acid concentrations, increased CD4^+^ and CD8^+^ T-cell counts, fewer markers of immune activation, and decreased levels of inflammatory cytokines than non-users (Manuzak et al., 2018). Together, these data provide a basis for the correlation between Δ^9^-THC usage and a lower incidence of HIV-associated neurocognitive disorders. It is therefore somewhat surprising that analyses into how, or even if, glial activation and senescence can be prevented using Δ^9^-THC as an adjunct to ART have not been completed.

**Delta9-tetrahydrocannabinol activates glia, independent of viral infection:** Δ^9^-THC is linked to activation of astrocytes, with varying effects depending on sex and age (Ramos-Jimenez et al., 2024). Through the binding of microglia and/or astrocyte-expressed CB2 Receptor (CB2R), a cascade of G-protein coupled receptor events exerts immunomodulatory effects on cytokine and presynaptic neurotransmitter release that ultimately alters neighboring extracellular environments (**Figure 1**). This could lead to a more pro-inflammatory phenotype if Δ^9^-THC is administered chronically prior to SIV infection, with varied effects in different brain regions depending on sex (Zamberletti et al., 2016). Additionally, the expression and function of endocannabinoid receptors are altered upon HIV infection, with CB_1_R upregulated in neurons and microglia and CB_2_R upregulated in microglia and astrocytes (Cosenza-Nashat et al., 2011). Putative mechanisms behind this feed-forward loop of inflammation include prolonged ART toxicity, persistent viral reservoirs within the CNS, and damage associated with the blood-brain barrier (Starr et al., 2021).

Beyond canonical receptor activation, the effects of Δ^9^-THC may also be modulated through heteromerization, a process increasingly recognized as a key regulator of neuroinflammatory signaling. For example, Δ^9^-THC signaling effects are potentiated by antagonism of the adenosine A_2A_ receptor (A_2A_R) in A_2A_R-CB_2_R heteromers, resulting in increased anti-inflammatory signaling in microglia (Rivas-Santisteban et al., 2025). Similarly, human epidermal growth factor receptor 2-CB_2_R complexes identified in glia and neurons link endocannabinoid signaling to cell proliferation and stress pathways (Blasco-Benito et al., 2019). These heteromeric interactions could represent an underexplored mechanism through which Δ^9^-THC dynamically rebalances pro- and anti-inflammatory cascades within the HIV-infected CNS. Investigating whether Δ^9^-THC stabilizes or disrupts such receptor complexes in astrocytes and microglia may yield new therapeutic insights.

Cannabinoids and viruses can independently enhance Toll-like receptor-mediated cytokine secretion in glia (Downer, 2011). This, of course, could also have pro- or anti-inflammatory consequences depending on which Toll-like receptors are expressed on the cells, those which are either up- or down-regulated, and finally on which agonists are present. Of recent interest, the senescence-associated secretory phenotype is an umbrella term for a heterogenous group of cytokines that are increased to different degrees depending on the mechanism of senescence induction. How Δ^9^-THC alters the senescence-associated secretory phenotype of multiple cell types in numerous conditions is a growing research topic.

In summary, Δ^9^-THC, when administered prior to infection, has been shown to decrease viral load in macaques infected with SIV. Very recent analyses are showing that Δ^9^-THC may prevent virus-induced senescence in the brain, including decreasing the senescence-associated secretory phenotype of glia and neurodegeneration. That said, chronic Δ^9^-THC use has also been associated with activation of many cell types, including leukocytes and glia. Taken together, Δ^9^-THC exerts a multifaceted influence in virus-induced neuroinflammation, capable of both dampening and amplifying glial activation depending on timing, dose, and cellular context. Future studies should move beyond descriptive analyses to address specific mechanistic questions. For example, does Δ^9^-THC alter the formation or stability of CB_2_R heteromers with A_2A_R or human epidermal growth factor receptor 2 in astrocytes and microglia of infected individuals? Also, could such complexes predict whether Δ^9^-THC treatment promotes neuroprotection versus neurotoxicity? To resolve these questions, researchers should employ spatial transcriptomics, single-cell multiomics, and super-resolution imaging in *in vitro* and *in vivo* models to map receptor colocalization and senescence-associated signaling. Additionally, longitudinal positron emission tomography imaging of CB2R availability in virally-suppressed primates could clarify whether Δ^9^-THC exposure correlates with regional neurodegeneration or cognitive resilience. By integrating these modern modalities, the field can finally define when Δ^9^-THC acts as a “double-edged sword” *versus* a “blunt weapon” against HIV-associated brain injury.

**Additional file:** *Open peer review report 1.*

OPEN PEER REVIEW REPORT 1
