# Supplementary material for: Delta9-tetrahydrocannabinol: A blunt weapon or a double-edged sword for virus-induced neuroinflammation
Source: Neural Regen Res. 2026 Jan 2;21(9):4237–8. doi: 10.4103/NRR.NRR-D-25-01394 (PMC13557690; doi:10.4103/NRR.NRR-D-25-01394)
Supplement: Supplementary file 1 [file NRR-21-4237_Suppl1.pdf]

OPEN PEER REVIEW REPORT 1

Name of journal: Neural Regeneration Research
Manuscript NO: NRR-D-25-01394
Title:
Reviewer's Name: Rafael Rivas-Santisteban
Reviewer's country: Spain

COMMENTS TO AUTHORS
This is a well-balanced "Perspective" article on the complex role of Δ^9-THC in virus-induced neuroinflammation, focusing on HIV/SIV. The manuscript effectively synthesizes the literature, highlighting Δ^9-THC's dual role as both potentially neuroprotective and pro-inflammatory. Therefore, I propose accepting the article after the following corrections are addressed.

1. Nomenclature Specificity: To be more precise and clarify that the discussion is exclusive to the delta-9 isomer, I recommend consistently using the abbreviation Δ9-THC instead of THC throughout the manuscript, including in the title.
2. Style and Abbreviations: Several abbreviations (e.g., HIV, CNS, ART) are used without being defined on their first appearance. Please ensure all abbreviations are defined. Additionally, use appropriate subscripts for receptor nomenclature (e.g., CB2 receptor or CB2R).
3. Discuss Receptor Heteromerization: The perspective could be significantly strengthened by discussing the potential for Δ9-THC to alter the formation of G-protein coupled receptor heteromers. For instance, cannabinoids are known to modulate the formation of heteromers like the A2AR-CB2R ((https://doi.org/10.1016/j.bcp.2025.117280) or HER2-CB2R (https://doi.org/10.1073/pnas.1815034116) complexes, which could be a relevant mechanism in neuroinflammation.
4. Specify Future Directions: The conclusion mentions the need for research using "modern modalities." To increase impact, please be more prescriptive. Suggest specific experimental questions that should be addressed in the near future to resolve the key unknowns highlighted in the perspective.
5. Revise the Figure: The schematic figure currently depicts Δ9-THC only as a protective agent that prevents astrocyte senescence. This does not fully represent the text, which also discusses Δ9-THC's potential to activate glia and promote inflammation. The figure should be modified to reflect this complexity, perhaps by adding an arrow to indicate this alternative pathway.
